# Retaining nurses in Sub-Saharan Africa: A systematic review and meta-analysis

**DOI:** 10.1016/j.ijnss.2025.04.004

**Published:** 2025-04-16

**Authors:** Evans Kasmai Kiptulon, Mohammed Elmadani, Mokaya Peter Onchuru, Anna Szőllősi, Miklós Zrínyi, Adrienn Ujváriné Siket

**Affiliations:** a Doctoral School of Health Sciences, Faculty of Health Sciences, University of Pécs, Pécs, Hungary; b The Kenya Medical Training College (Kapenguria Campus), Kapenguria, Kenya; c The County Government of West Pokot, Ministry of Health, Kapenguria, Kenya; d Doctoral School of Health Sciences, Faculty of Health Sciences, University of Debrecen, Debrecen, Hungary

**Keywords:** Intention to stay, Meta-analysis, Nurse, Retention, Sub-Saharan Africa, Systematic review

## Abstract

**Objectives:**

This study aimed to determine the current prevalence of nurse retention in Sub-Saharan Africa (SSA), evaluate the strategies and interventions in SSA countries used to retain their nurses, and identify the key challenges impeding nurse retention.

**Methods:**

A systematic review and meta-analysis were conducted. An electronic search was performed in August 2024 across multiple databases, including PubMed, Ovid Medline, Embase, CINAHL, Scopus, and grey literature sources. The studies were screened using Covidence, and quality assessments were conducted using the Mixed Methods Appraisal Tool.

**Results:**

A total of 31 articles were included in the review. Meta-analysis revealed that the pooled nurses’ retention rate in SSA was 53 % (95 %*CI*: 38 %–67 %; *I*^2^ = 97 %), while the pooled intention to stay (ITS) rate at work was 57 % (95 %*CI*: 43 %–71 %; *I*^2^ = 99 %). Subgroup analysis by region showed that the ITS rate was highest in East Africa (65 %), followed by West Africa (63 %), and lowest in Southern Africa (35 %). Effective retention strategies included financial and non-financial incentives, increased production and training of nurses, steering students to shortage specialties, adequate rural housing, facility level improvements, availability of career and professional progression opportunities, nurses’ recognition and involvement, employment terms, transparency and predictable management of human resources, supportive work environments, leadership, religious factors, and stakeholders’ collaborations. Key challenges to nurses’ retention include inadequate healthcare funding, governance issues, poor remuneration and working conditions, political interference, high unemployment rates, ineffective mobility management, unregulated international migration, and active recruitment by wealthier nations.

**Conclusions:**

Nurse retention in SSA remains critically low. Interventions should be formulated for the above-mentioned effective improvement strategies to address these systemic challenges in order to retain nurses in SSA.

## What is known?

• Sub-Sahara Africa (SSA) faces a critical nurse shortage, worsened by high turnover and mass migration to high-income countries.
• Retention strategies exist but lack region-specific effectiveness, and most previous systematic reviews and meta-analyses have mainly focused on high-income countries.

## What is new?

• This SSA-focused systematic review and meta-analysis revealed alarmingly low nurse retention (53 %) and intention-to-stay (57 %), with stark regional disparities.
• The review identified SSA-specific, unique, high-impact retention strategies while uncovering key challenges, offering actionable evidence that, if SSA countries implement, could strengthen nurse retention across the region.

## 1 Introduction

‘Retention of nurses’ is defined as the ability of a healthcare organization to keep its nursing staff employed over a prolonged time [1]. It involves maintaining a stable workforce by minimizing turnover and attrition rates, ensuring that nurses remain in their positions, and reducing the frequency of departures. Amidst the global shortage of nursing personnel and fast surging turnover and turnover intentions of nurses, the current top priority in the healthcare industry is the race to attract and retain the healthcare workforce, including nurses [2,3]. Nurse turnover, defined as nurses leaving an organization, resigning or terminating their careers [4], and turnover intention, which is the desire by nurses to leave their current position or organization [5], have become critical public health issues globally and more so for developing countries especially in Sub-Saharan Africa (SSA).

Africa is confronting a daunting public health crisis that demands immediate and decisive action to retain its medical personnel to achieve the 2030 Agenda and the Sustainable Development Goals [6], universal health coverage, and the aspirations of the African Agenda 2063 [7]. The mass exodus of medical experts, especially nurses, from SSA to high-income countries is a nursing and public health catastrophe that has continued to cripple, destabilize and weaken the already fragile healthcare system in the continent of Africa [8]. SSA, home to 18.3 % of the world population [9], bears 25 % of the global disease burden, endures the highest segment of the global maternal and neonatal mortality rates [10], and some of her sister countries face political instability [11]. The region also struggles with a high prevalence of infectious diseases such as HIV/AIDS, malaria, acute respiratory diseases, and tuberculosis, compounded by a fast-rising tide of non-communicable diseases like diabetes, hypertension, and cancer [12]. In addition, the region also suffers from recurrent disease outbreaks such as ebola, cholera, yellow fever, measles, zika virus, meningitis, and most recently, COVID-19 and monkeypox [13]. Health systems remain underfunded and understaffed, with inadequate infrastructure and limited access to essential medicines and critical healthcare workers [14]. Despite these immense problems, the SSA has only 3 % of the world 27.9 million nurses [15], accounts for 89 % of the five million global shortages of nurses [16], yet suffers from the highest nurse turnover and turnover intention rates globally. Recent studies in SSA have shown worryingly high turnover and turnover intention rates. In 2023, African nations experienced a loss of between 4,000 and 20,000 healthcare personnel to countries in Europe and America [17]. In the UK alone, for example, the National Health Service reported that over 48,677 healthcare workers of African origin were employed across the country in 2023. Among them were 22,851 Nigerians, 6,134 Ghanaians, 5,917 Zimbabweans, 4,148 Egyptians, 2,226 South Africans, and 1,936 Kenyans, along with others representing different African nationalities [18]. Similarly, in 2021, 13 % (364,000) of the 2.8 million immigrant healthcare workers in the USA were of African origin, with the majority being nurses [19]. African nurses have also immigrated to Canada, Australia, Germany, and other high-income countries. This trend only highlights, more than ever, the actual brain ‘gain’ versus brain ‘drain’ dynamics of mass health worker migration on the continent [17]. Under the current trajectory of nurse turnover and turnover intentions, and without immediate action, the three million shortages of nurses in SSA are predicted to double or triple by 2050 [15].

The significance of retaining the nursing workforce in small-economy countries like SSA cannot be overstated. High nurse turnover threatens fragile healthcare systems, economic progress, and a return on public investment in nurses’ training and production. Research suggests that Africa loses approximately US$ 2.0 billion annually due to the exodus of skilled healthcare professionals, including nurses [20]. Turnover increases healthcare costs, disrupts care, and leads to overwork, stress, and burnout among remaining staff, further driving turnover [21]. This cycle weakens healthcare delivery, increases medical errors, and hinders progress toward universal health coverage and the Sustainable Development Goals. Strengthening nurse retention is essential to ensure stable, effective healthcare systems.

While several systematic reviews [2,[22], [23], [24], [25], [26], [27], [28]] have been conducted on nurse retention strategies such as onboarding and early career interventions (externships, internships, extended orientation, transition-to-practice programs, nurse residency programs, special track programs), preceptorship, mentorship, and clinical ladder programs, these studies have predominantly focused on high-income countries. Other systematically well-researched methods include effective leadership, fostering a positive workplace culture, improving job satisfaction, offering competitive salaries, bonuses, and other incentives, providing career and professional advancement opportunities and social support, and integrating innovation and technology. Similarly, WHO [15,29] and other major international nursing organizations like ICN [30] have emphasized the urgent need to retain the nursing workforce in small nations, including SSA, through policy guidelines. Their suggested retention policy guides and methods are summarized in Appendix A. Although clear policy frameworks exist from WHO and ICN, nurse turnover in SSA remains critically high, highlighting a troubling reality: we still don’t know which strategies work in practice.

Despite a plethora of research on nurse retention, there remains a significant gap in the literature summarizing nurse retention in SSA. Limited systematic reviews have specifically addressed the unique challenges of the region. This study aimed to bridge this gap by analyzing a broad range of studies across 39 SSA countries that are included in the “WHO Health Workforce Support and Safeguards List 2023” [31] to identify the current prevalence of nurse retention, the effective country-specific retention strategies, challenges, and opportunities. This comprehensive synthesis will serve as a valuable resource for stakeholders, policymakers, researchers, and healthcare managers, guiding future research and helping to address the pressing issue of nurse retention and turnover in SSA.

## 2 Methods

### 2.1 Study design

This study was a systematic review and meta-analysis and followed the Preferred Reporting Items for Systematic Reviews and Meta-Analyses (PRISMA) [32]. Before we commenced the study, the protocol was registered in the International Prospective Register of Systematic Reviews (PROSPERO) with registration number CRD42024580615.

### 2.2 Search strategy

We used PubMed, Ovid Medline, Embase, CINAHL, and Scopus to search for the articles. Additionally, article searches were also performed using grey literature sources, including ResearchGate and Google Scholar, to supplement the major databases. The search terms included Nurs∗, nurses, nursing, Retain∗, retention, intention to stay, Angola, Benin, Burkina Faso, Burundi, Cameroon, Central African Republic, Chad, Comoros, Congo, Côte d’Ivoire, Democratic Republic of the Congo, Equatorial Guinea, Eritrea, Ethiopia, Gabon, Gambia, Ghana, Guinea, Guinea-Bissau, Lesotho, Liberia, Madagascar, Malawi, Mali, Mauritania, Mozambique, Niger, Nigeria, Rwanda, Senegal, Sierra Leone, South Sudan, Togo, Uganda, United Republic of Tanzania, Zambia, Zimbabwe, South Africa, and Kenya. The search encompassed the period from its inception to August 2024, and the detailed search strategy for each database is presented in Appendix B.

### 2.3 Inclusion and exclusion criteria

The inclusion criteria were as follows: 1) studies conducted in the 39 SSA countries [31]; 2) studies focusing on nurses of all cadres as the primary target population or mixed health care cadre studies, where nurses are the majority with distinct nurses’ data; 3) studies reported nurses’ retention/intention to stay (ITS) rates, or retention strategies/interventions (such as residency programs, leadership, salary, housing, education, and career opportunities, managing maldistribution, improved recruitment, mentorship, policy guidelines, etc) or key challenges impending nursing workforce retention; 4) qualitative, quantitative and mixed-methods studies; 5) studies in English. Only peer-reviewed articles published in the past 15 years were included. Conference papers or the full text that are not available were excluded.

### 2.4 Study selection

All articles retrieved through database searches were imported into EndNote for consolidation and deduplication. Subsequently, the articles were exported to Covidence software for further deduplication and screening. With Covidence’s blinding settings enabled, the authors (E.K. Kiptulon, M. Elmadani, M.P. Onchuru, and A. Szőllősi) conducted the initial title and abstract screening, followed by a full-text screening to assess the studies against the eligibility criteria. Disagreements at the two levels of screening were resolved through discussion and consensus.

### 2.5 Data extraction

The authors (E.K. Kiptulon, M. Elmadani, M.P. Onchuru, and A. Szőllősi) independently extracted data using a pre-designed and pre-tested standardized extraction form, which was agreed upon by the authors. Data collected from the included articles encompassed the following: primary author, year of publication, country of study, study design, sample size, study population, study setting, retention/ITS rates, retention interventions/strategies and challenges. Any discrepancies identified during this process were addressed and resolved through discussion and consensus.

### 2.6 Quality appraisal of the included studies

We performed quality appraisals for the included studies using the Mixed Methods Appraisal Tool (MMAT) [33]. The MMAT is a quality appraisal instrument designed to assess the methodological quality of five main study designs, including mixed methods, quantitative descriptive studies, non-randomized studies, randomized controlled trials, and qualitative research. The MMAT’s comprehensiveness and precision made it the ideal choice for assessing the diverse study designs included in our research. It consists of two parts: Part I is a checklist with two preliminary questions that must be passed to proceed to Part II. Part II contains five appraisal questions for each study design, scored as “Yes” (20 %) or “No/Can’t tell” (0). Each included study was evaluated based on the relevant criteria corresponding to its study type, ensuring a consistent and rigorous approach to quality assessment. To determine the quality of a study, the percentage scores from the five questions are averaged out of 100 %.

### 2.7 Data analysis

For the meta-analysis, we extracted the nurses’ retention or ITS rate (%) data as reported by the studies. Where articles reported data on scale, we transformed this data to percentage, and where data was presented in pre-post analysis, we obtained the average [34]. Using the MetaXL software, we conducted a pooled analysis using a weighted inverse variance random-effect model at a 95 %*CI* to determine the overall prevalence of nurses’ retention/ITS in their jobs. A sensitivity analysis was conducted to assess the stability of the meta-analysis results. We checked the heterogeneity of the publications using the *I*^2^ statistics and categorized values below 50 % as low and above 50 % as high. If heterogeneity was high, a random effect model was utilized; otherwise, a fixed effect model was employed. If sufficient data were available, subgroup analysis was conducted by region to explore the possible sources of heterogeneity. For the systematic review, we synthesized and critically analyzed results from included studies about the major grouping (Appendix A) of the WHO policy guidelines.

## 3 Results

**Fig. 1:**
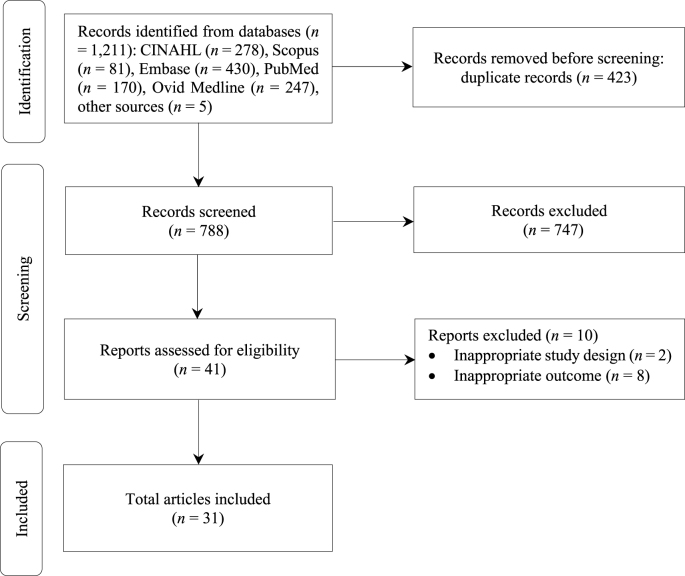
Flow chart of the study selection process.

The comprehensive search across all the databases identified 1,211 articles. Following a rigorous multi-step screening process, 31 articles were included (Fig. 1).

### 3.1 Characteristics of the included studies

All 31 included articles [[35], [36], [37], [38], [39], [40], [41], [42], [43], [44], [45], [46], [47], [48], [49], [50], [51], [52], [53], [54], [55], [56], [57], [58], [59], [60], [61], [62], [63], [64], [65]] were from 16 SSA states, with a majority of the articles (42 %, *n* = 13) being conducted in South Africa (*n* = 5), Ghana (*n* = 5), and Guinea (*n* = 3). Nigeria, Kenya, Malawi, Uganda, and Rwanda had two studies, and the Democratic Republic of the Congo, Zambia, the United Republic of Tanzania, Burkina Faso, Senegal, Cameroon, and Sierra Leone had one. Moreover, one article was conducted in Uganda and Zambia. Twelve (*n* = 12) studies were quantitative, while another 12 were qualitative, and seven articles were of mixed methods design. These studies were published between 2009 and 2024, with more than half (58 %, *n* = 18) published in the last five years. Most of the studies were conducted in hospitals and healthcare settings, with 10 conducted in remote and rural areas. Three studies were conducted among nursing students in nursing schools, 13 among professionally qualified nurses of different cadres, and 15 among mixed healthcare professionals, most of whom were nurses. The 31 articles had 30,972 study participants (Appendix C).

### 3.2 Quality of the included studies

The MMAT was used to assess the quality of the included studies. We found that all 31 articles were of high quality, with the lowest scoring of 80 % and the highest scoring of 100 % (Appendix D).

### 3.3 Prevalence of nurses’ retention and intention to stay in Sub-Saharan Africa

Three studies [46,59,61] reported the prevalence of nurses’ retention, while thirteen studies [35,38,[43], [44], [45],[50], [51], [52],^54^,^57^,^58^,^63^,^65^] reported ITS at work. The overall pooled nurses’ retention rate in SSA was 53 % (95 %*CI*: 38 %–67 %; *I*^2^ = 97 %) (Appendix E), while the pooled ITS rate at work was 57 % (95 %*CI*: 43 %–71 %; *I*^2^ = 99 %) (Appendix F). Sensitivity analysis results found that the pooled prevalence of the ITS rate remained stable, ranging from 54 % to 62 %, indicating that excluding any single study did not significantly affect the overall results. This stability underscores the robustness of our meta-analytic results. Severe heterogeneity was observed in the ITS rate analysis, and the number of studies was enough. Thus, we performed a subgroup analysis using region. Subgroup analysis results revealed that the ITS rate at work in SSA was the highest in East African at 65 %, followed by West African at 63 %, and lowest in the South Africa region at 35 % (Appendix F).

### 3.4 Strategies and interventions used by Sub-Saharan African countries to retain their nursing workforce

Appendix C presents the summary findings for the articles’ systematic review of the strategies and interventions used by SSA nations to retain their nursing workforce and the challenges faced. Our narrative synthesis grouped and examined these interventions based on the four major categories [29,66].

#### 3.4.1 Production – training and adapting the nursing workforce

This review conducted six studies [35,36,39,43,56,58] on nursing students within a training institution. The following were reported to be key interventions employed that play a role in retaining nurses in SSA.

##### 3.4.1.1 Production of nurses

Nursing production across countries in SSA has shown a consistent upward trend. In Kenya, the number of nursing students entering pre-service training rose significantly between 1999 and 2010, with annual admissions to pre-service nearly doubling from 1,493 in 1999 to 3,030 in 2010 [39]. Over this period, 23,350 students enrolled in nursing, reflecting a substantial commitment to expanding the nursing profession. A similar surge in nurse production is also witnessed across SSA nations, including Ghana, Nigeria, South Africa, Uganda, Tanzania, Cameroon, and Rwanda. WHO reported 2,772 nursing and midwifery institutions and a nursing workforce of 1,584,919 across Africa, marking a rise from 11.81 nurses per 10,000 people to 17.78 in 2020 [15]. This has created a pool of more nurses produced and retained in SSA.

##### 3.4.1.2 Steering students to shortage specialties and areas

Although the results of our review did not clearly show how SSA steered nursing production in specific nursing specialties like critical care/intensive care, emergency nursing, oncology, renal nursing, or psychiatric nursing, four studies [40,47,56,63] showed that SSA nations have focused mainly in community health nursing with targeted policies for the rural populace. These policies include recruiting from rural areas, providing rural training, and offering housing, career development, and conditional urban transfer incentives. This strategy aims to improve healthcare access in underserved areas by building a committed, community-oriented nursing workforce.

#### 3.4.2 Addressing inefficiencies and maldistribution of nurses: retention of nurses in the rural and remote regions of Sub-Saharan Africa

In this review, ten studies [47,48,50,51,53,55,56,61,64,65] were conducted in rural and remote SSA, and the following were found to be major reported interventions that retained nurses in rural settings.

##### 3.4.2.1 Financial and non-financial incentives

Both financial and non-financial incentives emerged to play a central role in the retention of the nursing workforce in rural [47,48,50,55,56,61,64]. Financial incentives include competitive salaries, risk, hardship, transport, transfer, and housing allowance, among other essential allowances. Okoroafor et al. [55], for example, found that healthcare workers (mainly nurses) were 2.7 times more likely (*OR* = 2.73; *P* ≤ 0.001) to take up rural posting and continue to stay if they receive a salary increment and four times more likely to take up rural posting (*OR* = 3.56; *P* ≤ 0.001) if housing allowance or a basic house is provided. Another study conducted in Cameroon found that student nurses were two times (a*OR* = 1.80; *P* < 0.001) more likely to take up rural posting and stay if a 75 % salary rural bonus is provided [56]. Non-financial incentives included professional recognition, subsidized education, and healthcare for nurses, among other incentives.

##### 3.4.2.2 Rural housing

Adequate housing emerged as a crucial retention factor for nurses in rural areas [55,56,65]. Accommodation that meets high comfort, privacy, and safety standards in rural and remote places can be challenging. Nurses are more retained with accommodations constructed by the government or employers for nurses and near the workplace. Berman et al. [65] found that nurses were 2.04 times more likely to choose and stay in rural jobs when superior housing is defined as a house with clean water, electricity, safety and security, good sanitation, internet, etc.

##### 3.4.2.3 Good physical facility/facility-level improvement

Retention was significantly influenced by the quality of facilities and the infrastructure [50,56,61,65]. Robyn et al. [56] found that student nurses were 3.5 times (a*OR* = 3.54; *P* ≤ 0.001) while qualified nurses were 3.6 times (a*OR* = 3.56; *P* < 0.001), more likely to take up rural posting and continue to stay if they there was a good hospital infrastructure, defined as a facility having staff housing, accessibility and connectivity to the city, availability of essential drugs, non-pharmaceuticals like personal protective equipment, medical equipment, adequate staff, and other resources.

##### 3.4.2.4 Training, career progression opportunities, and recognition

Opportunities for training, career advancement, and recognition significantly impacted retention [40,48,50,56,61,64]. Programs that allow for pre-service and in-service training [50], setting up nursing and midwifery schools in rural areas, recruiting rural students [40,56], and tailoring education to the specific needs of rural practice are essential to enhance retention. Although controversial, Twineamatsiko et al. [61] found that having a “certificate in nursing,” regarded as the lowest level of nursing training in Uganda, was associated with the retention of nurses. Manda et al. [48] found that career advancement opportunities, easy/flexible upgrading qualifications, opportunities to attend capacity building workshops/short training/seminars, certificates for acquiring new skills, networking, creating partnerships, and financial allowances boosted retention.

##### 3.4.2.5 Reduced cost of living and community support

Rural settings can often offer a reduced cost of living, which was found to be a retention factor for the nursing profession. Manda et al. [48] found that working in rural areas has a reduced cost of living, a supportive rural community, high regard, being valued and recognized, and easy access to farmland. Wurie et al. [64] similarly noted that these elements foster high levels of satisfaction with community service, creating a rewarding and revitalizing experience that strengthens nurses’ commitment to rural practice.

##### 3.4.2.6 High levels of nurses’ involvement

Active nurse involvement in hospital affairs and facility improvement strongly correlated with retention. A study conducted in rural districts of Rwanda [51] demonstrated that healthcare workers, including nurses, were nearly 100 times more likely to remain in their positions when they are highly involved in improving the quality of service within their hospitals (*OR* = 100.11; *P* = 0.001), six times more likely to remain when involved in the establishment of the hospital systems (*OR* = 6.01; *P* = 0.010) and overall, almost 11 times (*OR* = 10.95; *P* = 0.001) more likely to remain in the position when involved in every aspect of hospital affairs. These findings underscore that nurse involvement fosters a sense of ownership and professional satisfaction, which are critical to long-term retention.

##### 3.4.2.7 Type of employment: contract versus permanent

The nature of employment type, particularly the distinction between contractual and permanent employment, influenced retention [50,53]. Nurses with permanent employment types generally have more job security, career stability, and access to loans from financial institutions for personal development, among other diverse opportunities that encourage longer tenures.

##### 3.4.2.8 Fair, transparent, and predictable management of human resources by the ministry of health

A Predictable and transparent human resource management system by the Ministry of Health fosters a stable work environment. This includes clear policies on transfers, promotions, and other human resources-related matters, which offer nurses a sense of security regarding their career progression and location stability [47,50,56]. A study from Cameroon Robyn et al. [56] found that nursing students were three times (a*OR* = 2.81; *P* < 0.001) while qualified nurses were 2.3 times (a*OR* = 2.31; *P* < 0.001) more likely to choose a rural job and stay if they are assured of guaranteed transfer after service. Nagai et al. [50] and Kouanda et al. [47] found that fair, predictable, and well-implemented HR policies are associated with increased satisfaction and retention.

##### 3.4.2.9 Religious factors

Religious factors also contribute to retention, especially where religious beliefs and practices align with the work environment. A study from Senegal [50] found that family bonding and religious factors were special factors in retaining nurses and the healthcare workforce. Workplaces that accommodate or respect religious practices can increase job satisfaction and commitment among staff, indirectly contributing to retention.

#### 3.4.3 Improving recruitment and retention of nurses

Apart from the above factors identified to enhance retention in rural and remote SSA, other major general retention factors are as follows.

##### 3.4.3.1 Supportive work environment

A supportive work environment is pivotal for nurse retention. Several studies [[42], [43], [44], [45],^48^,^57^,^58^,^61^,^62^,^64^,^65^] identified work environment characteristics including flexible work schedules, adequacy of staff and resources, good interpersonal relationships, positive organizational culture, safety and security at workplace, equipment, and supplies, being respected and valued, manageable workloads, organizational and leadership support and good physical facility as factors that retained nurses in different countries in SSA.

##### 3.4.3.2 Leadership

Leadership and management were found to play a critical role in retaining nurses in SSA. A study conducted in Rwanda [52] found that directive, supportive, and participative leadership styles significantly explained 38 %, 10 %, and 23 % of the variance in job satisfaction, ITS, and service provision, respectively. Likewise, in Ghana, participative, transformational, and transactional leadership styles significantly explain 20.9 % of the variance in ITS [54]. Empowering nurse managers through training on leadership, leadership roles, management skills, managerial attributes, and managerial roles in retaining a multi-generational workforce was emphasized by South Africa’s nurse managers as vital in retaining the nursing workforce. Supportive supervision and manager support further strengthened retention efforts in SSA [61,63].

##### 3.4.3.3 Professional and career development

Professional development is the continuous process of acquiring new knowledge, skills, and competencies to enhance performance and effectiveness. In contrast, career development involves long-term planning and opportunities for growth, job group advancement, and achieving broader career goals. Several studies [42,48,58,61,62] found that professional and career development was essential for nurse retention in SSA.

##### 3.4.3.4 Other retention methods

The other identified retention methods include improved remuneration [[43], [44], [45],^50^,^55^,^56^,^60^,[62], [63], [64]], re-attraction of retired and overseas nurses [42], government policy [40,42,47], collaboration between government and donor stakeholders [60] and improving image of nursing [42].

#### 3.4.4 Managing mobility and flow of nurses out of Sub-Saharan Africa

Despite the WHO, ICN, and other stakeholders’ emphasis on efficient nurse mobility management through monitoring movements, fostering bilateral agreements, and reintegrating foreign-trained nurses, our review found no SSA evidence demonstrating structured approaches to tackling nurse retention. While South-African nurse managers have highlighted the re-attraction of retired and overseas nurses [42], the absence of documented practices across other SSA nations suggests a significant gap, potentially reflecting a lack of coordinated efforts or actionable policies addressing this critical issue.

### 3.5 Challenges faced by Sub-Saharan Africa countries in retaining nursing workforce

#### 3.5.1 Problems in production and training

SSA faces significant barriers in nurse production and training, as evidenced by our included studies from Ghana [36,37], Guinea [62], and Kenya [39]. These challenges include limited training capacity, limited clinical mentorship, lack of knowledge of rotation objectives by clinical staff, inadequate supervision and support of students in clinical rotations, congestion due to insufficient classroom space, lack of accommodation facilities for students, inadequate transportation, congestion at clinical areas, student attrition, overproduction versus high unemployment, some training curriculum does not align with Ministry of Health needs, poor collaboration between ministry of health and ministry of finance in training, lack of lecturers and difficulty in retaining faculty staff, lack of transparency in recruitment after graduation and low pay for nurses.

#### 3.5.2 Governance issues, leadership, and political interference

We found that poor policy implementation, poor funding of healthcare, unclear governance structure between national and local/county (lower tiers) governments, political interference, and compromised autonomy of nurses hinder nurses’ retention [47,49,53,59]. In Nigeria, for example, Nwankwo et al. [53] found salary disparities in different tiers of government, inadequate resources, political interference where politicians focus more on infrastructure and buildings at the expense of healthcare workforce development, political interests overriding professional policymakers, concentration of health facilities in urban areas, lower tiers of government being prone to policy alteration, contractual employment types, low and delayed salaries, and poor human resource planning. In Kenya, Shikuku 2022 et al. [59] found that in county governments (lower tier governments), there was high unprofessional regard to education and specialization transfers that affected the retention of healthcare workers after undergoing emergency obstetric and newborn care training.

#### 3.5.3 Poor work environment

Inadequate medical supplies and equipment, shortage of nurses, high patient ratios, poor workplace safety, work-related stress, burnout, adverse patient outcomes, litigations, strained interpersonal, inter-cadre, and inter-profession relationships, inflexible work schedules, lack of recognition, inadequate organizational and managerial support, limited accommodation and transport facilities, job insecurity, increased utilization of temporary/contractual employment terms, rural stigma, poor social amenities in rural and too much paperwork were identified as key challenges related to the poor work environment [41,44,49,53,58,62,64].

#### 3.5.4 International migration and wealthier global northern countries

Our review found that rich and powerful nations offer irresistible higher salaries, better working conditions, and career advancement opportunities abroad, making it challenging to retain nurses in Africa [43].

## 4 Discussion

This study represents the comprehensive and dedicated systematic review and meta-analysis on nurses’ retention in SSA. It was conducted in 37 SSA countries in the WHO’s “Health Workforce Safeguards List 2023” [31]. These nations are also featured on the UK’s National Health Service red list, with Kenya (due to its previous placement on the amber list) and South Africa (due to high turnover reports) being the only two countries included outside the “Health Workforce Safeguards List 2023”. Each country faces severe health workforce shortages and vulnerabilities, with a Universal Health Coverage Service Coverage Index below 50 and a physician, nurse, and midwife density significantly below the global median of 48.6 per 10,000 people [31,67,68]. Our review revealed alarmingly low rates of nurses’ retention and ITS in SSA, with pooled prevalence rates of 53 % and 57 %, respectively. The study further identified that ITS at work in SSA was the highest in East Africa at 65 %, followed by West Africa at 63 %, and lowest in the South Africa region at 35 %. These results were similar to those of previous studies [16,29,[69], [70], [71], [72], [73]], which have found similarly poor retention and high turnover of nurses in SSA. Sub-group analysis from this study showed that retention of nurses in SSA was highest in East Africa, followed by West Africa, and lowest in the South Africa region. This slightly contrasts the findings by Ayalew et al. [69], who found that turnover intention among nurses in SSA was highest in East Africa (58 %) and lowest in the Southern Africa region (33 %), with an overall rate of 50 % [69]. The analysis of the Southern African region was predominantly based on studies from a single country: the Republic of South Africa. The lower ITS in Southern Africa may be attributed to increased workloads, working overtime, poor quality of midwifery care, low morale, high levels of work-related stress and burnout, lack of training opportunities, increased utilization of temporarily employed midwives, and increased international recruitment of nurses from South Africa by high-income countries [43,49]. In addition, ITS differences within SSA regions could be due to socio-economic differences, government policies, salary differences, cost of living, and the ease of immigration. SSA bears the highest disease burden and has only 3 % of the global nursing workforce, so these findings underscore a deeply concerning workforce challenge.

Our review identified several practical and effective retention strategies within WHO 2010/2020 guidelines that have been implemented by SSA nations as *production*; increased nurses’ production through training and steering students to shortage specialties [40,48,50,56,61,64], *addressing insufficiencies and maldistribution with a focus on rural and remote SSA;* financial and non-financial incentives [47,48,50,55,56,61,64], rural housing [55,56,65], rural facility infrastructural improvement [50,56,61,65], opportunities for career and professional progression, community support, high levels of nurses' involvement, opportunities for training and recognition [40,48,50,56,61,64], permanent employment terms [42,48,58,61,62], predictable management of human resource and religious respect [47,50,56]*, improving recruitment and retention;* supportive work environments [[42], [43], [44], [45],^48^,^57^,^58^,^61^,^62^,^64^,^65^], supportive, participatory, transformative, and transactional leadership [52,61,63], professional and career development opportunities [42,48,58,61,62], supportive government policies [[42], [43], [44], [45],^48^,^57^,^58^,^61^,^62^,^64^,^65^]. These findings align with other systematic reviews such as [2,16,24,69,[74], [75], [76], [77], [78], [79], [80]] on variables that enhance nurses’ retention. However, strategies to manage nurse mobility and mitigate outflows of nurses from SSA were poorly implemented or lacked sufficient data to assess their effectiveness. The study differed from other systematic reviews done in higher-income countries such as [2,[22], [23], [24], [25], [26], [27]] and [28], which have alluded that externships, internships, extended orientation, transition-to-practice programs, nurse residency programs, special track programs), preceptorship, mentorship, and clinical ladder programs remain primary methods to retain the nursing workforce. These stark differences highlight a critical divergence in approach: in high-income settings, retention strategies are embedded early, starting during nursing school, and are designed to support the individual nurse continuously throughout their professional journey. This proactive and sustained engagement is the cornerstone of workforce stability in those contexts. In contrast, such structured, longitudinal retention mechanisms are underutilized in SSA. This disparity underscores a missed opportunity for SSA countries to adopt early intervention models that prepare student nurses for practice and anchor them in the profession over the long term.

Furthermore, several challenges complicating the retention of the nursing workforce in SSA were identified as issues related to the production and training of nurses, such as limited capacity, poor mentorship, and misaligned curricula, among other academic challenges; this could reflect internal systemic weaknesses. In addition, governance issues like poor policy implementation [53] and funding gaps stem from broader structural political dysfunctions in SSA nations. While training problems hinder workforce entry, poor work environments characterized by staff shortages, high workloads, and inadequate resources—drive existing nurses to leave. Unlike the strategic nature of governance and training issues, these are operational daily stressors. These pull and push factors have also been raised worldwide, not only in SSA [[81], [82], [83]]. International migration adds a contrasting global dimension. Unlike internal push factors, migration is driven by external pull: better pay, conditions of work, and career opportunities abroad [43,70,78,84]. Together, these challenges create a cycle of poor retention fueled by local failures and global inequities.

To retain the nursing workforce in SSA, this study recommends permanent employment terms for nurses, improved salary, supportive work environments, robust professional and career development opportunities, adequate production of nurses, flexible work schedules, adequacy of staff and resources-equipment and supplies, good interpersonal relationships at the workplace, increased involvement of nurses in hospital affairs, positive organizational culture and climate, enhancing safety and security at the workplace, transparent and predictable management of the human resource, organizational and leadership support, improved image of nursing, professional autonomy, housing, community engagement and involvement, respecting nurses’ religious affiliations, improving rural facilities, and collaboration between stakeholders. Furthermore, the WHO and the International Labor Organization must enforce binding agreements to protect SSA nursing resources, ensuring ethical recruitment and compensation for source countries. A strict “employ-one, train-one” policy against wealthy nations recruiting nurses from SSA should be non-negotiable. This will curb the exploitation of the fragile nations in SSA by more affluent countries. Bilateral agreements between SSA and high-income nations must mandate reinvestment of resources into local health systems, such as funding nursing education, infrastructure, and professional development. The World Bank, International Monetary Fund, and global donors must prioritize workforce sustainability in SSA by earmarking funds for competitive salaries, career development, rural healthcare incentives, and expanding nursing education, including free nursing and subsidized education. Digital platforms should be developed to provide virtual training, mentorship, and remote career progression opportunities, reducing the need for migration in pursuit of professional growth. Lastly, the African Union must establish a region-wide nursing protection policy ensuring harmonized employment terms, salary benchmarks, and career progression structure through its regional blocs like the Economic Community of West African States, East African Community, and the Southern African Development Community. Implementation of regional licensing and certification recognition to facilitate intra-African mobility while preventing excessive immigration to high-income nations. All SSA nations must decisively enforce WHO's recommendations for fully retaining their nursing workforce.

## 5 Study limitations

Results from this study should be interpreted with caution due to some limitations. First is the high heterogeneity among the included studies, which reflects variability in study designs, study populations, retention variables, and methods, potentially affecting the generalization of the findings. There was also limited regional representation, with the included studies coming from only 16 SSA countries, mainly from eastern, western, and southern regions of Africa. This means, therefore, that some contexts and challenges may have been underrepresented. Furthermore, the studies were mainly published in English; hence, potential information in other languages like French, Portuguese, and German, which are common in SSA, might have been missed. In addition, our study primarily relied on peer-reviewed articles and indexed databases, which excluded unpublished reports and government documents, potentially leading to publication bias. Finally, the study focused on retention and ITS metrics, leaving other factors like burnout, job satisfaction, and external socio-economic influences. Addressing these gaps in future research, including incorporating multilingual literature, particularly in French and Portuguese, common languages in Africa, may provide a more comprehensive understanding of nurse retention in SSA.

## 6 Conclusions

This review found that nurses’ retention in SSA was critically low (53 %), with ITS at 57 %, reflecting a severe workforce crisis. Effective retention interventions included financial and non-financial incentives, increased production and training of nurses, steering students to shortage specialties, rural housing, facility level improvements, availability of career and professional progression opportunities, nurses’ recognition and involvement, employment terms, transparency and predictable management of human resources, supportive work environments, leadership, religious factors, and stakeholders’ collaborations. Challenges persist at multiple levels, including inadequate healthcare funding, governance issues, poor remuneration and working conditions, political interference, high unemployment rates, ineffective mobility management, unregulated international migration, and active recruitment by wealthier nations. To move forward, SSA nations must fully implement WHO recommendations, invest in long-term retention strategies, and establish regional policies to harmonize employment conditions. Global bodies must enforce ethical recruitment and support SSA through funding, capacity building, and binding bilateral agreements. Without decisive action, the region risks further depleting its fragile nursing workforce.

## Data availability statement

The datasets generated during and/or analyzed during the current study are available from the corresponding author upon reasonable request.

## CRediT authorship contribution statement

**Evans Kasmai Kiptulon:** Conceptualization, Methodology, Validation, Formal analysis, Investigation, Data curation, Writing - original draft. **Mohammed Elmadani:** Conceptualization, Methodology, Validation, Formal analysis, Investigation, Resources, Data curation, Writing - review & editing. **Mokaya Peter Onchuru:** Conceptualization, Methodology, Validation, Formal analysis, Investigation, Resources, Data curation, Writing - review & editing. **Anna Szőllősi:** Methodology, Validation, Formal analysis. **Miklós Zrínyi:** Conceptualization, Methodology, Validation, Formal analysis, Investigation, Resources, Data curation, Writing - review & editing, Supervision, Project administration. **Adrienn Ujváriné Siket:** Conceptualization, Methodology, Validation, Formal analysis, Investigation, Resources, Data curation, Writing - review & editing, Supervision, Project administration.

## Declaration of competing interests

There is no conflict of interests in this study.
