# Supplementary material for: Retaining nurses in Sub-Saharan Africa: A systematic review and meta-analysis
Source: Int J Nurs Sci. 2025 Apr 16;12(3):301–9. doi: 10.1016/j.ijnss.2025.04.004 (PMC12168464; doi:10.1016/j.ijnss.2025.04.004)
Supplement: Multimedia component 1 [file mmc1.docx]

**撒哈拉以南非洲地区护士的留任及提升策略：系统评价和meta分析**

Evans Kasmai Kiptulon，Mohammed Elmadani，Mokaya Peter Onchuru，Anna Szőllősi，Miklós Zrínyi，Adrienn Ujváriné Siket

## ****【摘要】****

### ****目的**** 明确撒哈拉以南非洲（Sub-Sahara Africa，SSA）地区护士的留任率及意愿，汇总其提升策略和干预措施以及面临的挑战。

### ****方法**** 计算机检索PubMed、Ovid Medline、Embase、CINAHL、Scopus和灰色文献，检索时限为建库至2024年8月。使用Covidence进行文献筛选，用混合方法评价工具进行质量评估。

### ****结果**** 共纳入31篇文献。Meta分析结果显示，SSA地区护士留任率为53%（95%*CI*: 38% ~ 67%, *1*^2^=97%），留任意愿为57%（95%*CI*: 43% ~ 71%, *1*^2^=99%）。亚组分析结果表明东非护士（65%）的留职意愿最高，其次是西非（63%），南非最低（35%）。提升护士留任的有效策略包括财政和非财政激励：增加护士的培养和培训、引导护生进入短缺专科领域及欠发达地区、充足的农村住房、改善医疗设施状况、提供职业发展机会、加强护士参与度、尊重宗教信仰、支持性工作环境、领导力培训以及利益相关者之间的密切合作等。护士留住的主要挑战包括培训能力弱与条件差、医保资金投入不足、招聘过程不透明、管理问题、待遇和工作条件差、政治性干预、护士自主权不高、职业倦怠、人际关系紧张、国际移民开放政策以及高收入国家更具吸引力的招聘条件等。

### ****结论**** SSA地区的护士留任率及意愿较低，需结合上述有效提升策略并联合多方力量制订有针对性的干预措施，以解决所面临的严峻挑战，从而提高护士的留任。

## ****【关键词】**** 留任意愿；Meta分析；护士；留任；撒哈拉以南非洲；系统评价

**通信作者：**Evans Kasmai Kiptulon，E-mail：evans.kasmai.kiptulon@pte.hu
