# Supplementary material for: Retaining nurses in Sub-Saharan Africa: A systematic review and meta-analysis
Source: Int J Nurs Sci. 2025 Apr 16;12(3):301–9. doi: 10.1016/j.ijnss.2025.04.004 (PMC12168464; doi:10.1016/j.ijnss.2025.04.004)

Appendix A Summary of policy guidelines (based on WHO 2010 & 2020 nurse retention for small countries).

| Policy area of action | Options may include |
| --- | --- |
| Production – training  and adapting the nursing  workforce | Transforming the education of the nursing workforce: continuous professional development; re-skilling; redefining skills in line with population needs; lifetime learning; steering students to shortage specialties and areas; broadening out the recruitment base by targeting underrepresented groups; investing in education capacity; adapting curricula to demography and disease profiles; harnessing technology. |
| Better managing mobility  and flows of nurses | Monitoring flows; bilateral agreements; integration of foreign-trained nurses and international returnees. |
| Improving recruitment and retention of nurses | Creating supportive and safe workplaces; flexible working hours; professional autonomy; professional development and career progression; expansion of roles; remuneration; return to practice; retraining/additional training. |
| Addressing inefficiencies and maldistribution of nurses | Financial and non-financial incentives; education; regulation; professional and personal support; harness technology; performance management; skill mix changes and new roles. |

Appendix B Search strategy of each database.

| Databases | Search strategy | No. of articles |
| --- | --- | --- |
| PubMed | #1 ((nurs*[Title/Abstract]) OR (nurses [Title/Abstract])) OR (nursing [Title/Abstract]) | 197,620 |
|  | #2((Retain*[Title/Abstract]) OR (retention [Title/Abstract])) OR (‘intention to stay’[Title/Abstract]) | 199,756 |
|  | #3 ((((((((((((((((((((((((((((((((((((((Africa[Title/Abstract]) OR (Angola[Title/Abstract])) OR (Benin[Title/Abstract])) OR (Burkina Faso[Title/Abstract])) OR (Burundi[Title/Abstract])) OR (Cameroon[Title/Abstract])) OR (Central African Republic[Title/Abstract])) OR (Chad[Title/Abstract])) OR (Comoros[Title/Abstract])) OR (Congo[Title/Abstract])) OR (Côte d’Ivoire[Title/Abstract])) OR (Democratic Republic of the Congo[Title/Abstract])) OR (Equatorial Guinea[Title/Abstract])) OR (Eritrea[Title/Abstract])) OR (Ethiopia[Title/Abstract])) OR (Gabon[Title/Abstract])) OR (Gambia[Title/Abstract])) OR (Ghana[Title/Abstract])) OR (Guinea[Title/Abstract])) OR (Guinea-Bissau[Title/Abstract])) OR (Lesotho[Title/Abstract])) OR (Liberia[Title/Abstract])) OR (Madagascar[Title/Abstract])) OR (Malawi[Title/Abstract])) OR (Mali[Title/Abstract])) OR (Mauritania[Title/Abstract])) OR (Mozambique[Title/Abstract])) OR (Niger[Title/Abstract])) OR (Nigeria[Title/Abstract])) OR (Rwanda[Title/Abstract])) OR (Senegal[Title/Abstract])) OR (Sierra Leone[Title/Abstract])) OR (South Sudan[Title/Abstract])) OR (Togo[Title/Abstract])) OR (Uganda[Title/Abstract])) OR (United Republic of Tanzania[Title/Abstract])) OR (Zambia[Title/Abstract])) OR (Zimbabwe[Title/Abstract])) OR (South Africa[Title/Abstract])) OR (Kenya[Title/Abstract]) | 480,292 |
|  | #4= ((#1) AND (#2)) AND (#3) Filters: in the last 15 years, English | 170 |
| Ovid Medline | \|  \| #1“nurs*”.ab,ti \| \| --- \| --- \| | 547,540 |
|  | #2 nurses.ab,ti. | 231,576 |
|  | #3 nursing. ab,ti. | 309,804 |
|  | #4 "Retain*”. ab,ti. | 257,775 |
|  | #5 Retention.ab,ti. | 228,465 |
|  | #6 ‘intention to stay’. ab,ti. | 255 |
|  | #7= (#1 or #2 or #3) | 547,540 |
|  | #8= (#4 or #5 or #6) | 46,304 |
|  | #9(Africa or Angola or Benin or Burkina Faso or Burundi or Cameroon or Central African Republic or Chad or Comoros or Congo or Ivory coast or Democratic Republic of the Congo or Equatorial Guinea or Eritrea or Ethiopia or Gabon or Gambia or Ghana or Guinea or Guinea-Bissau or Lesotho or Liberia or Madagascar or Malawi or Mali or Mauritania or Mozambique or Niger or Nigeria or Rwanda or Senegal or Sierra Leone or South Sudan or Togo or Uganda or Republic of Tanzania or Zambia or Zimbabwe or South Africa or Kenya).ab,ti. | 262,268 |
|  | #10= (#7 AND #8 AND #9) | 353 |
|  | limit 10 to (english language and yr="2014 - 2024") | 247 |
| Scopus | ( TITLE-ABS-KEY ( nurs* OR nursing OR nurses ) AND TITLE-ABS-KEY ( retain* OR retention OR "intention to stay" ) AND TITLE-ABS-KEY ( africa OR angola OR benin OR "Burkina Faso" OR burundi OR cameroon OR "Central African Republic" OR chad OR comoros OR congo OR ivory AND coast OR "Democratic Republic of the Congo" OR "Equatorial Guinea" OR eritrea OR ethiopia OR gabon OR gambia OR ghana OR guinea OR "Guinea-Bissau" OR lesotho OR liberia OR madagascar OR malawi OR mali OR mauritania OR mozambique OR niger OR nigeria OR rwanda OR senegal OR "Sierra Leone" OR "South Sudan" OR togo OR uganda OR "Republic of Tanzania" OR zambia OR Zimbabwe OR South Africa OR kenya ) ) AND PUBYEAR > 2013 AND PUBYEAR < 2025 | 81 |
| Embase | #1 nurs* | 3,880,150 |
|  | #2 nurses | 349,664 |
|  | #3 nursing | 3,653,856 |
|  | #4= (#1 OR #2 OR #3) | 3,880,150 |
|  | #5 retain* | 310,268 |
|  | #6 retention | 344,846 |
|  | #7 ‘intention to stay’ | 243 |
|  | #8= (#5 OR #6 OR #7) | 625,502 |
|  | #9 ((((((((africa OR angola OR benin OR burkina) AND faso OR burundi OR cameroon OR central) AND african AND republic OR chad OR comoros OR congo OR ivory) AND coast OR democratic) AND republic AND of AND the AND congo OR equatorial) AND guinea OR eritrea OR ethiopia OR gabon OR gambia OR ghana OR guinea OR ‘guinea bissau’ OR lesotho OR liberia OR madagascar OR malawi OR mali OR mauritania OR mozambique OR niger OR nigeria OR rwanda OR senegal OR sierra) AND leone OR south) AND sudan OR togo OR uganda OR republic) AND of AND tanzania OR zambia OR zimbabwe OR OR South Africa OR kenya | 98,767 |
|  | #10 = (#4 AND #8 AND #9) | 468 |
|  | #11 = (#10 AND (2014:py OR 2015:py OR 2016:py OR 2017:py OR 2018:py OR 2019:py OR 2020:py OR 2021:py OR 2022:py OR 2023:py OR 2024:py)) | 430 |
| CINAHL | "(nurses or nurses or nursing ) AND ( retain employees or retention or intention to stay’ or ) AND ( africa OR banin OR burkina OR burkina) AND fa benso OR burundi OR cameroon OR cameroon OR koror or ivory OR (or coast OR ivory OR) AND republic of the AND and con-congoghana OR guinea OR ‘guinea bissau’ OR lesotho OR liberia OR madagascar OR malawi OR mai OR mauritania OR niger OR niger OR rigeria OR rhuna OR sierra) AND leone OR south) AND sudan OR togo OR uganda OR uganda OR) AND of tanzania OR zambia OR Zimbabwe OR South Africa OR Kenya ) | 278 |

Appendix C Characteristics of the included studies (*n* = 31).

| Study | Country | Study design | Study population | Sample | Study setting | Retention/ITS rate (%) | Retention interventions/strategies and the challenges |
| --- | --- | --- | --- | --- | --- | --- | --- |
| Abugri et al., 2018 [35] | Ghana | Quantitative | Student nurses | 220 | Nursing school | ITS score = 3.68 ± 1.14 | Positive attitudes increased with the intent to stay in nursing.  Low pay, lack of travel opportunities and view of nursing as a challenging career increased ITS. |
| Adatara et al., 2022 [36] | Ghana | Qualitative | New graduate nurses | 30 | Hospital | - | Acquisition of more knowledge to be a professional nurse was strongly noted.  Clinical staff lacked knowledge of rotation objectives, frustrations, inadequate supervision and support, lack of accommodation and financial challenges were the main challenge. |
| Adatara et al., 2023 [37] | Ghana | Qualitative | Frontline nurses | 15 | Hospital | - | Physical and emotional challenges, inadequate information and training during the initial stages of COVID-19, inadequate staffing and logistics, stigmatization, and exclusion from the frontline with its benefits. |
| Agyapong et al., 2015 [38] | Ghana | Quantitative | Mental health workers 43 % of nurses | 204 | Mental health unit | ITS rate = 71.8 % | Stigma, job risks, lack of supervisor and organizational support, lack of respect, limited professional development, poor working conditions i.e. low salary, inadequate facilities, transport, accommodation, poor allowances, and strained interpersonal relationships. |
| Appiagyei et al., 2014 [39] | Kenya | Mixed method | Student nurses | 23,350 | Nursing school | Retention rate = 96 % | Congestion in clinical areas, limited clinical mentorship, difficulty in recruitment and retaining faculty staff, inadequate accommodation facilities for students, inadequate transport, classroom space and student attrition. |
| Baba et al., 2020 [40] | Democratic Republic of the Congo | Qualitative | Healthcare workers | 49 | Healthcare (workshop) | - | Most attraction and retention policies were not implemented or only partially implemented. Proposed strategies include starting rural midwifery classes, recruiting rural students, promoting health service use to generate income, and seeking support from NGOs and churches |
| Mtega et al., 2017 [41] | Tanzania | Qualitative | Registered nurses | 5 | Public hospital | - | Nurses’ shortage driven by high patient ratios, results in delayed patient care, increased ITS, adverse patient outcomes, increased workplace injuries and reduced patient satisfaction. Retention strategies include hiring more nurses, improving professional development opportunities, and increasing incentives. |
| Mokoka et al., 2010 [42] | South Africa | Qualitative | Nurse managers | 21 | Public and private hospitals | - | General factors: improving working conditions, flexible working hours, professional development, rewards and benefits, interpersonal relationships at the workplace, re-attracting retired and overseas nurses, improving the image of nursing, values and organizational culture.  Organizational factors: work safety, inadequate resources, organizational policy and strategic planning, organizational culture and change. Nurse manager and leadership roles: empower nurse managers, training and development on leadership roles, management skills, managerial attributes, and managerial role in retaining a multi-generational workforce. |
| Zieand & Liang 2024 [43] | South Africa | Quantitative | Nursing students | 536 | Hospital | ITS rate = 50 % | The main motivators for nurses’ immigration are higher salaries, better working conditions and career advancement opportunities abroad. Significant demographic factors are age, marital status, and family responsibility. |
| Mokoka et al., 2011[44] | South Africa | Quantitative | Registered nurses | 108 | Hospital | ITS rate = 26.9 % | The decision to stay in the current job by over 90 % of the nurses was determined by finances, safety and security, equipment and supplies, management, staff and patient ratio. Reviewing and improving salary, annual bonuses, improved security and safety, availing equipment and supplies and enhancing staff-patient ratios could strongly retain nurses. |
| Kim et al., 2021[45] | Uganda & Zambia | Quantitative | Health-care providers (96 %) nurses | 1,134 | Hospital | ITS rate = 42.8 % | Demographic determinants: gender, cadre, facility resources, country. Facility/Management factors: satisfaction with pay/salary, being respected at the workplace, manageable workloads and adequate number of staff. Lack of clinical education affected the intention to stay. |
| Kolie et al., 2021 [46] | Guinea | Mixed method | Healthcare workers-nurses (26 %) | 611 | Healthcare | Retention rate = 41 % for nurses | The Ebola program successfully recruited and distributed healthcare workers, positively improving healthcare delivery in rural areas. However, challenges arose, including healthcare workers’ unfamiliarity with primary healthcare delivery, staff conflicts, and issues related to a high number of female recruits. Absenteeism was high, driven by unknown factors, continuing education, illness and maternity leave. Governance and management issues, such as unclear roles between national and local authorities, highlighted the need for clear policy guidance. |
| Kouanda et al., 2014 [47] | Burkina Faso | Qualitative | Healthcare workers | 70 | Hospital remote areas | - | The regionalized policy was introduced in response to uneven distribution, rural vs urban. *The policy implementation (2002-2003):* Recruitment to the rural regions without transfers. In 2005, opened for job-to-job transfers and a competitive transfer process afterwards. *Policy issues:* lack of written directives, targeting one category of personnel, no financial/non-financial incentives. |
| Manda et al., 2023 [48] | Zambia | Qualitative | Healthcare workers | 28 | Rural health facilities | - | Professional development factors: career advancement opportunities, easy upgrading qualification, opportunities to attend capacity building workshops/ short training/seminars, a certificate for acquiring new skills, networking, creating partnerships and financial allowances. Work environment factors: challenging tasks, promotions, co-worker recognition, and supportive relationships. Rural community and working in rural: reduced cost of living, community support and recognition, easy access to farmland. |
| Matlala et al., 2019 [49] | South Africa | Qualitative | Midwives | 11 | Public hospitals | - | Shortages: increased workloads, working overtime, poor quality of midwifery care, low morale, work-related stress and burnout, lack of training opportunities, increased utilization of temporarily employed midwives. Management-related issues for leaving the profession: lack of management support, fear of litigations, finances, no recognition, compromised autonomy, too much paperwork, and lack of flexibility on schedule.  Reasons for staying professional and personal limitations- fear for change, passion for midwifery, no other place to go, availability for training and other opportunities. |
| Nagai et al., 2017 [50] | Senegal | Qualitative | Healthcare workers | 176 | Rural healthcare facilities | ITS rate = 46 % | Motivating/demotivating factors: pre-service and in-service training, regulatory system, financial and non-financial incentives and environmental support. Factors outside WHO recommendation found highly important: fairness, transparency and predictable management of human resources by MoH, employment type permanent versus contract. Non-financial family bonding and religion were found special factors. |
| Ndikumana et al., 2019[51] | Rwanda | Quantitative | Healthcare workers | 252 | Public district hospitals | ITS rate = 51.63 % for nurses | Workers who perceived a high level of involvement: in improving the quality of service in the hospital were 100 times more likely to stay (*OR* = 100.111; *P* = 0.001), involvement in establishments of systems (*OR* = 6.005; *P* = 0.010). Overall involvement increased the likelihood of stay by almost 11 times (*OR* = 10.952; *P* = 0.001) |
| Ngabonzima et al., 2020 [52] | Rwanda | Quantitative | Nurses & midwives | 162 | Hospitals | ITS score = 3.42 ± 0.77 | Directive, supportive and participative leadership styles were dominant. All leadership styles together significantly explained 38 %, 10 % and 23 % of the variance in job satisfaction, intention to stay and service provision, respectively. |
| Nwankwo et al., 2022 [53] | Nigeria | Qualitative | Healthcare workers | 23 | Rural hospitals | - | Socio-cultural factors: rural stigma, with urban seen as symbolic of higher status. Health system factors: salary disparities in different tiers of government, inadequate resources, political interference-focus on infrastructure and building at the expense of the healthcare workforce, political interests overriding professional policymakers, concentration of health facilities in urban, decentralization with lower tiers of government prone to policy alteration, contractual employment types, low and delayed salaries, poor human resource planning. Intrinsic Health worker’s factors: isolation, family impact, personal perception and beliefs, personal characteristics |
| Ofei & Paarima 2022 [54] | Ghana | Quantitative | General nurses | 348 | Hospitals | ITS rate = 85.6 % | Participative, transformational and transactional leadership styles were dominant. All leadership styles together significantly explained 20.9 % of the variance in intention to stay among nurses. |
| Okoroafor et al., 2022 [55] | Nigeria | Mixed method | Healthcare workers | 198 | Rural hospitals | - | Healthcare workers were 2.7 (*OR* = 2.73; *P* ≤ 0.001) times more likely to take up rural posting and continue to stay if they receive a salary increment and four times more likely to take up rural posting (*OR* = 3.56; *P* ≤ 0.001) if housing allowance or a basic house is provided. |
| Robyn et al., 2015 [56] | Cameroon | Discrete choice experiment/Mixed method | Students & Healthcare workers | 351 | Healthcare facilities | - | Student nurses were 3.5 times (a*OR* = 3.54; *P* ≤ 0.001) more likely to take up rural posting and continue to stay if they get good hospital infrastructure (defined as having staff housing, accessibility and connectivity to city, availability of drugs, equipment, adequate staff, opportunities for career development, guaranteed transfer to urban after fixed period), three times more like to stay (a*OR* = 2.81, *P* < 0.001) if assured of guaranteed transfer, two times (a*OR* = 1.80, *P* < 0.001) more likely if 75 % salary bonus is provided and brought up in rural (a*OR* = 1.68, *P* = 0.049). For qualified nurses, good hospital (aOR=3.56, *P* < 0.001), salary bonus (a*OR* = 3.13, *P* < 0.001) and guaranteed transfer to urban (a*OR* = 2.31, *P* < 0.001). |
| Rubin Pillay 2009 [57] | South Africa | Quantitative | General nurses | 569 | Hospital | ITS rate =30.2 % | Younger nurses and working in rural were less likely to stay in their jobs. Of those intending to stay, employment security, workplace organization and positive work environment were major contributing factors. |
| Schmiedeknecht et al., 2015 [58] | Malawi | Mixed method | Nurse graduates of a scholarship | 86 | Hospital | ITS rate (average) =80 % | Motivators to continue work: job security, desire to continue further education, public service agreement  Push factors: inadequate supplies, inadequate staff, high workloads, poor co-worker/management working relationships, low salary, lack of housing and lack of appreciation/recognition, |
| Shikuku et al., 2022 [59] | Kenya | Quantitative | Health workers (73 %, nurses & midwives) | 927 | Hospital obstetric unit | Retention rate = 46 % | One year after the training, only 36 % of the trained staff were found retained in the specialized ward/unit (emergency obstetric and newborn). Unprofessional without regard to specialization transfers in the devolved governments (counties governments). Transfer policy should be strengthened. |
| Taderera et al., 2016 [60] | Zimbabwe | Quantitative | Health workers | 101 | Health facilities | - | Collaborative partnership between the government and donor community enhanced retention. Government: reviewed salaries, supported training and development and provided protection. Donor: offered top-up allowances and non-financial incentives. |
| Twineamatsiko et al., 2023 [61] | Uganda | Quantitative | Health workers | 235 | Remote public health centers | Retention rate = 71.49 % | Individual factors associated with retention: Having a certificate as the highest academic level, staying with family, and working more than 6 years in the facility. Health system factors: good physical facility, availability of equipment, flexible work schedules, adequate staff, organizational and manager support, being valued and respected and incentives. Career Factors: job satisfaction, job motivation, promotions and opportunities for further studies |
| Pas et al., 2019 [62] | Guinea | Mixed method | Health workers | 57 | Hospitals | - | Policy position on retention: localize recruitment, strengthen supervision, improved salaries, community involvement, career development, improving the work environment, and financial/non-financial incentives. Noted Challenges: overproduction vs limited employment, many volunteered in the hope of being employed, MoH’s poor management of the health workforce, lack of transparency in recruitment, training curriculum does not align with MoH needs, poor collaboration between Ministry of Health and Ministry of finance in training. |
| Witter et al., 2021[63] | Guinea | Survey | Health workers | 600 | Healthcare facilities | ITS rate (average) = 13 % | There was high satisfaction with supervision and non-financial incentives. The financial aspects were found least satisfactory and a high proportion of staff consider emigration. Proposed policy l for retention: increased recruitment from rural, fixed work time contract in rural, improving work conditions, actionable incentive package, offering upgrading and specialization in return to rural service, training and decentralization of human resource funding. |
| Wurie et al., 2016 [64] | Sierra Leone | Qualitative | Health workers | 23 | Rural health facilities | - | Motivating retention factors: satisfaction with community service, role effectiveness, financial incentives, and training opportunities. Challenges: difficult terrain, rural living with poor social amenities, poor working conditions, emotional and financial costs of separation from family, limited training opportunities, longer working hours, limited career progression, inadequate financial/non-financial incentives, political interference in recruitment etc, and strained relationships with local community. Policy recommendation: financial/non-financial incentives, rural staff housing, mobility and communication, training opportunities and career progression, improved working conditions, social amenities, separation allowance and relocation package and transparent fair procedures. |
| Berman et al., 2021 [65] | Malawi | Mixed method | Nurse midwives | 472 | Rural hospitals | ITS rate (average) = 59.3 % | Housing, facility-level improvement, management, improved work environment and workload were key factors in nurses’ attraction and retention. Participants were 2.04 times more likely to choose a rural job with superior housing and 1.70 times more likely with better facility quality. |

*Note:* ITS = Intention to stay. NGOs = Non governmental organization. MoH = Ministry of Health.

Appendix D Quality appraisal results of the included studies.

| Study | Appraisal questions | | | | | | | | | | | | | | | | | | | | | | | | | |
| --- | --- | --- | --- | --- | --- | --- | --- | --- | --- | --- | --- | --- | --- | --- | --- | --- | --- | --- | --- | --- | --- | --- | --- | --- | --- | --- |
|  | 1.1 | 1.2 | 1.3 | 1.4 | 1.5 | 2.1 | 2.2 | 2.3 | 2.4 | 2.5 | 3.1 | 3.2 | 3.3 | 3.4 | 3.5 | 4.1 | 4.2 | 4.3 | 4.4 | 4.5 | 5.1 | 5.2 | 5.3 | 5.4 | 5.5 | Scale |
| Abugri et al., 2018 [35] |  |  |  |  |  |  |  |  |  |  |  |  |  |  |  | Y | Y | Y | Y | Y |  |  |  |  |  | 100% |
| Adatara et al., 2022 [36] | Y | Y | Y | Y | Y |  |  |  |  |  |  |  |  |  |  |  |  |  |  |  |  |  |  |  |  | 100% |
| Adatara et al., 2023 [37] | Y | Y | Y | Y | Y |  |  |  |  |  |  |  |  |  |  |  |  |  |  |  |  |  |  |  |  | 100% |
| Agyapong et al., 2015 [38] |  |  |  |  |  |  |  |  |  |  |  |  |  |  |  | Y | Y | Y | Y | Y |  |  |  |  |  | 100% |
| Appiagyei et al., 2014 [39] |  |  |  |  |  |  |  |  |  |  |  |  |  |  |  |  |  |  |  |  | Y | Y | Y | CT | Y | 80% |
| Baba et al., 2020 [40] | Y | Y | Y | Y | Y |  |  |  |  |  |  |  |  |  |  |  |  |  |  |  |  |  |  |  |  | 100% |
| Mtega et al., 2017 [41] | Y | CT | Y | Y | Y |  |  |  |  |  |  |  |  |  |  |  |  |  |  |  |  |  |  |  |  | 80% |
| Mokoka et al., 2010 [42] | Y | Y | Y | Y | Y |  |  |  |  |  |  |  |  |  |  |  |  |  |  |  |  |  |  |  |  | 100% |
| Zieand & Liang 2024 [43] |  |  |  |  |  |  |  |  |  |  |  |  |  |  |  | Y | Y | Y | N | Y |  |  |  |  |  | 80% |
| Mokoka et al., 2011 [44] |  |  |  |  |  |  |  |  |  |  |  |  |  |  |  | Y | Y | Y | CT | Y |  |  |  |  |  | 80% |
| Kim et al., 2021 [45] |  |  |  |  |  |  |  |  |  |  |  |  |  |  |  | Y | Y | Y | Y | Y |  |  |  |  |  | 100% |
| Kolie et al., 2021 [46] |  |  |  |  |  |  |  |  |  |  |  |  |  |  |  |  |  |  |  |  | Y | Y | Y | Y | Y | 100% |
| Kouanda et al., 2014 [47] | Y | Y | Y | CT | Y |  |  |  |  |  |  |  |  |  |  |  |  |  |  |  |  |  |  |  |  | 80% |
| Manda et al., 2023 [48] | Y | Y | Y | Y | Y |  |  |  |  |  |  |  |  |  |  |  |  |  |  |  |  |  |  |  |  | 100% |
| Matlala et al., 2019 [49] | Y | Y | Y | Y | Y |  |  |  |  |  |  |  |  |  |  |  |  |  |  |  |  |  |  |  |  | 100% |
| Nagai et al., 2017 [50] | Y | Y | Y | Y | Y |  |  |  |  |  |  |  |  |  |  |  |  |  |  |  |  |  |  |  |  | 100% |
| Ndikumana et al., 2019 [51] |  |  |  |  |  |  |  |  |  |  |  |  |  |  |  | Y | Y | Y | Y | Y |  |  |  |  |  | 100% |
| Ngabonzima et al., 2020 [52] |  |  |  |  |  |  |  |  |  |  |  |  |  |  |  | Y | Y | Y | Y | Y |  |  |  |  |  | 100% |
| Nwankwo et al., 2022 [53] | Y | Y | Y | Y | Y |  |  |  |  |  |  |  |  |  |  |  |  |  |  |  |  |  |  |  |  | 100% |
| Ofei & Paarima 2022 [54] |  |  |  |  |  |  |  |  |  |  |  |  |  |  |  | Y | Y | Y | Y | Y |  |  |  |  |  | 100% |
| Okoroafor et al., 2022 [55] |  |  |  |  |  |  |  |  |  |  |  |  |  |  |  |  |  |  |  |  | Y | Y | Y | CT | Y | 80% |
| Robyn et al., 2015 [56] |  |  |  |  |  |  |  |  |  |  |  |  |  |  |  |  |  |  |  |  | Y | Y | Y | Y | Y | 100% |
| Rubin Pillay 2009 [57] |  |  |  |  |  |  |  |  |  |  |  |  |  |  |  | Y | Y | Y | Y | Y |  |  |  |  |  | 100% |
| Schmiedeknecht et al., 2015 [58] |  |  |  |  |  |  |  |  |  |  |  |  |  |  |  |  |  |  |  |  | Y | Y | Y | Y | Y | 100% |
| Shikuku et al., 2022 [50] |  |  |  |  |  |  |  |  |  |  |  |  |  |  |  | Y | Y | Y | Y | Y |  |  |  |  |  | 100% |
| Taderera et al., 2016 [60] | Y | Y | Y | Y | Y |  |  |  |  |  |  |  |  |  |  |  |  |  |  |  |  |  |  |  |  | 100% |
| Twineamatsiko et al., 2023 [61] |  |  |  |  |  |  |  |  |  |  |  |  |  |  |  | Y | Y | Y | Y | Y |  |  |  |  |  | 100% |
| Pas et al., 2019 [62] |  |  |  |  |  |  |  |  |  |  |  |  |  |  |  |  |  |  |  |  | Y | Y | Y | N | Y | 80% |
| Witter et al., 2021 [63] |  |  |  |  |  |  |  |  |  |  |  |  |  |  |  | Y | Y | Y | CT | Y |  |  |  |  |  | 80% |
| Wurie et al., 2016 [64] | Y | Y | Y | Y | Y |  |  |  |  |  |  |  |  |  |  |  |  |  |  |  |  |  |  |  |  | 100% |
| Berman et al., 2021 [65] |  |  |  |  |  |  |  |  |  |  |  |  |  |  |  |  |  |  |  |  | Y | Y | Y | CT | Y | 80% |

*Note:* 1.1: Is the qualitative approach appropriate to answer the research question? 1.2: Are the qualitative data collection methods adequate to address the research question? 1.3: Are the findings adequately derived from the data? 1.4: Is the interpretation of results sufficiently substantiated by data? 1.5: Is there coherence between qualitative data sources, collection, analysis and interpretation? 2.1: Is randomization appropriately performed? 2.2: Are the groups comparable at baseline? 2.3: Are there complete outcome data? 2.4: Are outcome assessors blinded to the intervention provided? 2.5: Did the participants adhere to the assigned intervention? 3.1: Are the participants representative of the target population? 3.2: Are measurements appropriate regarding both the outcome and intervention (or exposure)? 3.3: Are there complete outcome data? 3.4: Are the confounders accounted for in the design and analysis? 3.5: During the study period, is the intervention administered (or exposure occurred) as intended? 4.1: Is the sampling strategy relevant to address the research question? 4.2: Is the sample representative of the target population? 4.3: Are the measurements appropriate? 4.4: Is the risk of nonresponse bias low? 4.5: Is the statistical analysis appropriate to answer the research question? 5.1: Is there an adequate rationale for using a mixed methods design to address the research question? 5.2: Are the different components of the study effectively integrated to answer the research question? 5.3: Are the outputs of the integration of qualitative and quantitative components adequately interpreted? 5.4: Are divergences and inconsistencies between quantitative and qualitative results adequately addressed? Y = yes. N = no. CT = cannot tell.

Appendix E Forest plot for pooled nurses’ retention in Sub-Sahara Africa (n = 3).


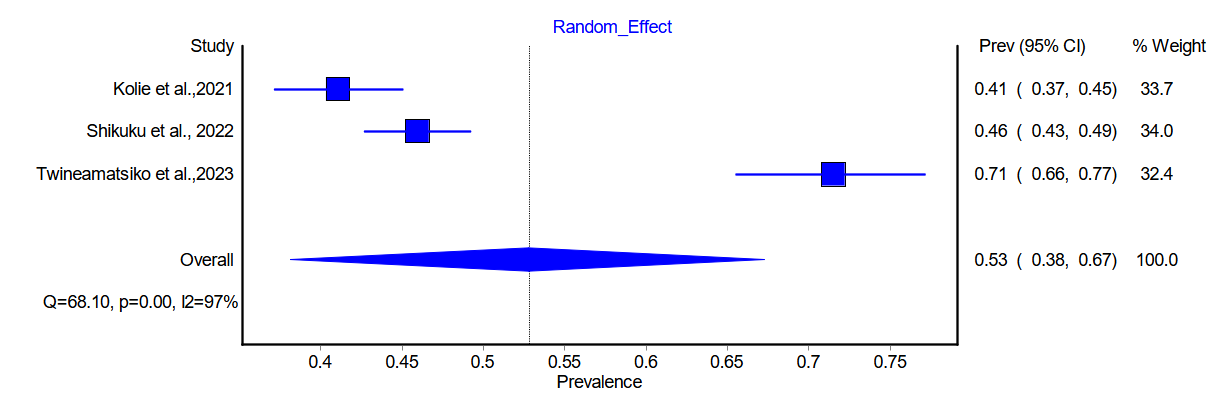


Appendix F Forest plot showing nurses’ intention to stay at work in Sub-Sahara Africa (*n* = 13).


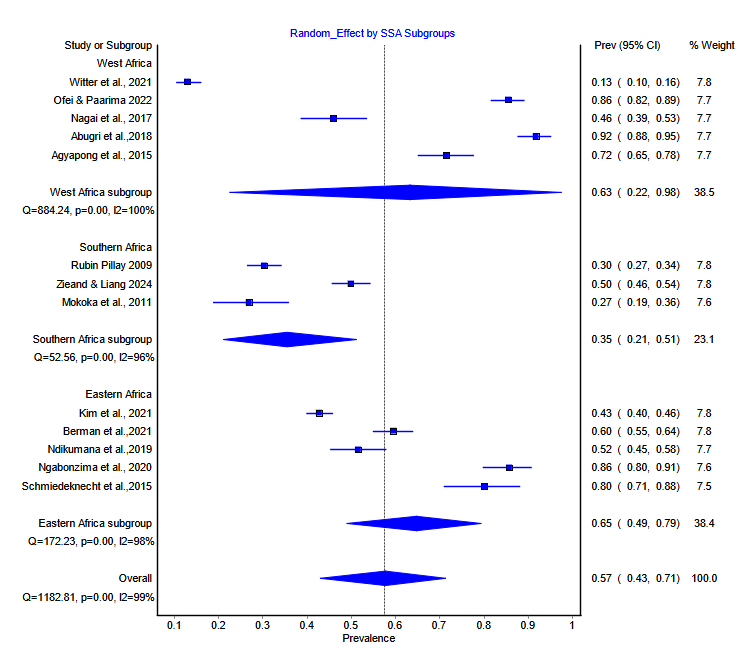

Supplement: Multimedia component 2 [file mmc2.docx]
